# Supplementary material for: Inflammatory factors and risk of lung adenocarcinoma: a Mendelian randomization study mediated by blood metabolites
Source: Front Endocrinol (Lausanne). 2024 Aug 27;15:1446863. doi: 10.3389/fendo.2024.1446863 (PMC11384989; doi:10.3389/fendo.2024.1446863)
Supplement: Supplementary file 1 [file DataSheet1.zip › supplementary files/S2/GCST90201000/forest.pdf]

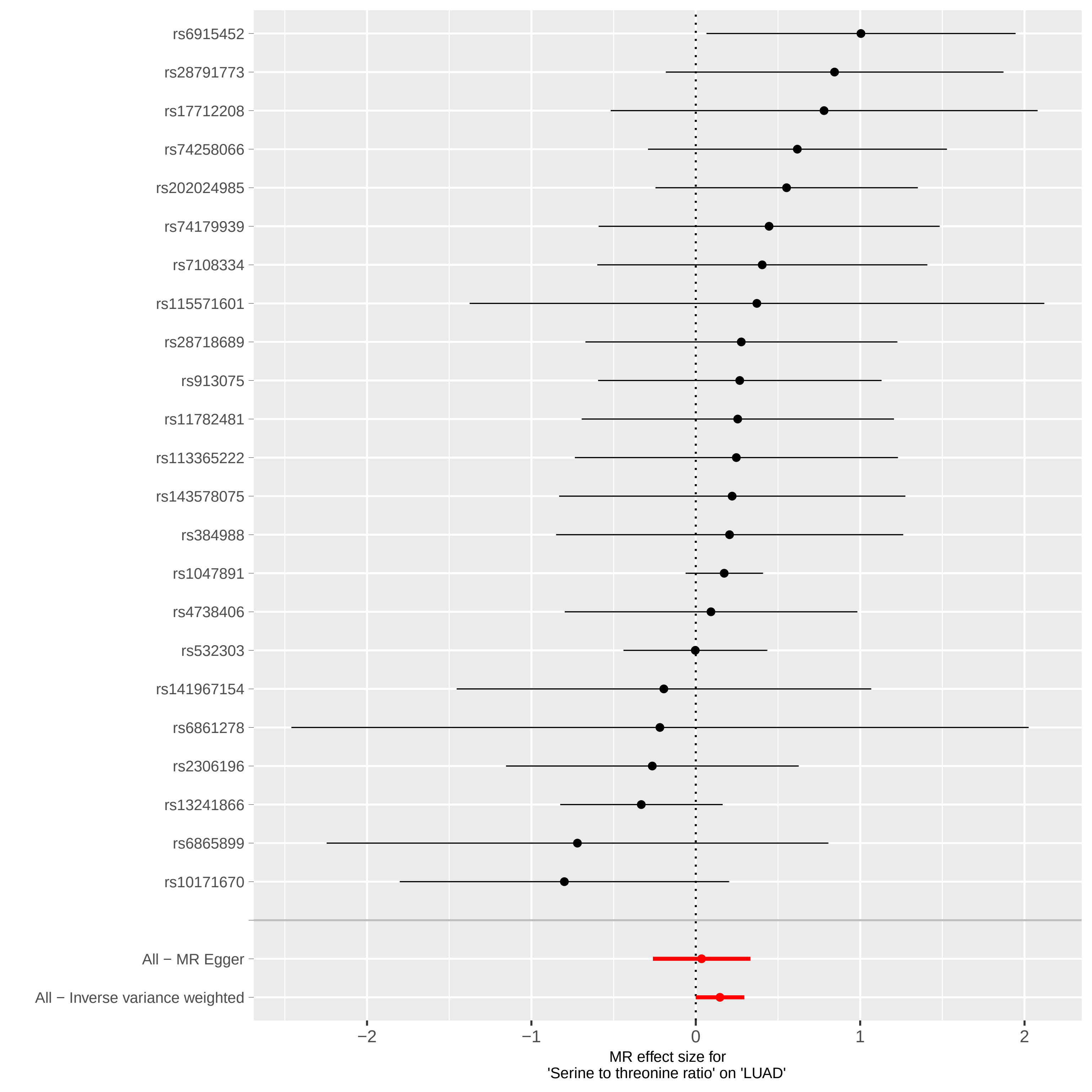
rs6915452
rs28791773
rs17712208
rs74258066
rs202024985
rs74179939
rs7108334
rs115571601
rs28718689
rs913075
rs11782481
rs113365222
rs143578075
rs384988
rs1047891
rs4738406
rs532303
rs141967154
rs6861278
rs2306196
rs13241866
rs6865899
rs10171670
All − MR Egger
All − Inverse variance weighted
−2
−1
0
1
2
MR effect size for
'Serine to threonine ratio' on 'LUAD'
